# Adalimumab Ameliorates Abdominal Aorta Cross Clamping Which Induced Liver Injury in Rats

**DOI:** 10.1155/2014/907915

**Published:** 2014-01-16

**Authors:** Erkan Cure, Medine Cumhur Cure, Levent Tumkaya, Yildiray Kalkan, Ibrahim Aydin, Aynur Kirbas, Arif Yilmaz, Suleyman Yuce, Ahmet Fikret Yücel

**Affiliations:** ^1^Department of Internal Medicine, School of Medicine, Recep Tayyip Erdogan University, 53100 Rize, Turkey; ^2^Department of Biochemistry, School of Medicine, Recep Tayyip Erdogan University, 53100 Rize, Turkey; ^3^Department of Histology and Embryology, School of Medicine, Recep Tayyip Erdogan University, 53100 Rize, Turkey; ^4^Department of Surgery, School of Medicine, Recep Tayyip Erdogan University, 53100 Rize, Turkey; ^5^Department of Gastroenterology, School of Medicine, Recep Tayyip Erdogan University, 53100 Rize, Turkey

## Abstract

The aim of this study was to investigate the possible protective effects of adalimumab (ADA) on cell damage in rat liver tissue during ischemia/reperfusion (I/R) injury of infrarenal abdominal aorta. Thirty male Wistar-albino rats were divided into three groups: control, I/R, and I/R+ADA, each group containing 10 animals. Laparotomy without I/R injury was performed in the control group animals. Laparotomy in the I/R group was followed by two hours of infrarenal abdominal aortic cross ligation and then two hours of reperfusion. ADA (50 mg/kg) was administered intraperitoneally as a single dose, to the I/R+ADA group, five days before I/R. The tumor necrosis factor-alpha (TNF-*α*) (pg/mg protein) and nitric oxide (NO) (*µ*mol/g protein) levels in the I/R group (430.8 ± 70.1, 8.0 ± 1.1, resp.) were significantly higher than those in the I/R+ADA group (338.0 ± 71.6, *P* = 0.006; 6.3 ± 1.2, *P* = 0.008) and the control group (345.5 ± 53.3, *P* = 0.008; 6.5 ± 1.5, *P* = 0.010, resp.). I/R causes severe histopathological injury to the liver tissue, but ADA leads to much less histopathological changes. ADA treatment significantly decreased the severity of liver I/R injury. ADA pretreatment may have protective effects on experimental liver injury.

## 1. Introduction

Hepatic ischemia/reperfusion (I/R) injury affects the prognosis of patients in a vast clinical range, including transplantation, liver resection surgery, trauma and hemorrhagic shock, and aortic injury during abdominal surgery [1]. While aortic occlusion is carried out, the blood supply is occluded to organs such as the liver. The obstruction of the aorta and consequent reperfusion leads to distant organ injury via multiple mechanisms including neutrophilic infiltration, the production of reactive oxygen species (ROS), the release of cytokines such as the tumor necrosis factor-alpha (TNF-*α*) and elevation of nitric oxide (NO) levels [2–4]. Also, decreased arginase and carbamoyl phosphate synthetase-1 (CPS-1) enzyme activities are associated with tissue injury by elevated NO levels [5–8]. Reperfusion injury occurs after permitting blood reflow into an ischemic tissue, and the surge of oxygen to low oxygenated tissues causes an increased production of ROS [9]. The apoptosis pathway is activated as a result of mitochondrial damage due to increased ROS. Apoptosis plays a major role in liver injury induced by I/R [10].

TNF-*α* is a pleiotropic cytokine that has biological effects ranging from cell death to inducing tissue regeneration [11–13]. TNF-*α* is released at the beginning of reperfusion, and its level increases during the early phases of I/R [14]. The inhibition of TNF-*α* release, or its neutralization with anti-TNF-*α* antibodies, decreases the number of neutrophils infiltrating the liver, reducing liver I/R injury [15]. Adalimumab (ADA), which is the first fully human monoclonal antibody targeted against TNF-*α*, was first administered to study patients in 1997. It has been reported that it can be safely used for certain diseases [16]. Previous studies have reported that infliximab, which is another inhibitor of TNF-*α*, can also decrease/prevent the damage of TNF-*α* in I/R models [17, 18].

The twofold aim of this study is to determine whether the inhibition of TNF-*α* ameliorates I/R-induced liver tissue injury by suppressing cell damage and whether the inhibition of TNF-*α* alters the NO balance by its effect on arginase and CPS-1 activity in liver I/R injury.

## 2. Materials and Methods

### 2.1. Animals

Thirty Wistar-albino male rats, weighing 250–300 g (12–15 weeks old), were used in the present study. The rats were indiscriminately divided into three groups: control group (*n* = 10), I/R group (*n* = 10), and I/R+ADA group (*n* = 10). This research was performed in accordance with the Guide for the Care and Use of Laboratory Animals (NIH, 1985) and approved by the local ethical committee at the Medical School of the Recep Tayyip Erdogan University (Approval numbers: 2012/11).

### 2.2. Experimental Design

The rats in the control and I/R groups received saline solution. The control group underwent a midline laparotomy and dissection of the infrarenal abdominal aortic cross (IAA) without obstruction. The I/R group underwent laparotomy and clamping of the IAA for 120 minutes, followed by 120 minutes of reperfusion. ADA (Humira; Abbott, Abbott Park, Ill) (40 mg/0.8 mL) was diluted in saline and given as one bolus applied in an intraperitoneal single dose injection of 50 mg/kg to the I/R+ADA group [19]. After five days of ADA application, the I/R+ADA group underwent 120 minutes of ischemia and 120 minutes of reperfusion.

### 2.3. Aortic I/R

The I/R model was designed in a way similar to previous studies [2, 20]. The rats were anesthetized with ketamine hydrochloride (50 mg/kg intramuscularly) (Ketalar; Eczacibasi, Istanbul, Turkey), and anesthesia was maintained with supplementary intramuscular injections of ketamine hydrochloride. The rats were located in a supine position under a heating lamp. The skin was prepared aseptically, and a midline laparotomy was implemented. Warm normal saline (10 mL) was dribbled into the peritoneal cavity to help maintain the fluid balance. The abdominal aorta was exposed by politely deflecting the loops of the intestine to the left with splashy gauze materials. An atraumatic microvascular clamp was located across the IAA. The abdomen was switched off, and the wound was covered with plastic wrap to minimize the loss of heat and fluid. After 120 minutes, the microvascular clamp on the IAA was removed and the lower limb reperfusion was maintained for 120 minutes. Aortic occlusion and reperfusion were corroborated by the loss and resurrection of the pulsation on the distal aorta; therefore, a no-reflow phenomenon was excluded. At the end of the reperfusion, a median sternotomy was enforced, and blood samples were drawn from the right ventricles of all rats for biochemical analyses. All rats were euthanized under anesthesia and their livers were cautiously removed. The specimens were stored for further biochemical and histological analyses.

### 2.4. Biochemical Parameters

Blood samples (10 mL) were taken from all rats and collected into routine tubes to evaluate the biochemical tests. The blood was separated by centrifugation @ 3,000 rpm for 10 min, after standing at room temperature for 15 min. The biochemical parameters, including activities of serum enzymes aspartate aminotransferase (AST), alanine aminotransferase (ALT), and concentrations of urea and creatinine were determined in the serum by using commercial kits (ARCHITECT C16000, Abbott Laboratories, USA).

#### 2.4.1. Tissue Homogenates

After weighing the liver tissues, they were homogenized in ten volumes of ice-cold Phosphate Buffer Saline (PBS) (50 mM, pH 7.4) and centrifuged at 10,000 xg for 20 minutes. The homogenization procedure (wiseTise homogeniser, Korea) was carried out for 2 min at 10,000 rpm. All procedures were performed at 4°C. Aliquots of the supernatant were put into tubes and frozen at −80°C. Homogenate, supernatant, and extracted samples were prepared and the following determinations were made on the samples using commercial chemicals (Sigma, St. Louis, MO, USA). All the parameters were checked within one month.

#### 2.4.2. Measurement of Protein

The tissue homogenate protein assay is a turbidimetric procedure in which benzethonium chloride is used as the protein denaturing agent. Proteins in the form of a fine suspension were quantitated turbidimetrically at 404 *η*m (ARCHITECT c16000, Abbott Laboratories, USA).

#### 2.4.3. Tissue TNF-*α*

The concentration of TNF-*α* was measured using an enzyme-linked immunosorbent assay (ELISA) method with a commercially available rat TNF-*α* ELISA kit (eBioscience, Vienna, Austria). The absorbance was measured at a *λ* of 450 *η*m, using the ELISA reader. The intra-assay and interassay coefficients of variation were <5% and <10%, respectively. The limit of detection (LOD) for the TNF-*α* assay was 11 pg/mL. When dividing the obtained values by the protein levels, the final results were obtained as pg/mg protein.

#### 2.4.4. Tissue NO

The concentrations of NO were measured using the colorimetric assay method, with a commercially available ELISA kit (Cayman Chemical Company, USA). The absorbance was measured at a *λ* of 540 *η*m using the reader. The intra-assay and interassay coefficients of variation were 2.7% and 3.4%, respectively, and the LOD for the NO assay was 2.5 *μ*M. When dividing the obtained values by the protein levels, the final results were obtained as *μ*mol/g protein.

### 2.5. Immunohistological Evaluation

For immunohistochemical staining 3-4 *μ*m thick sections of the liver tissues were cut and allowed to stand in xylene for 20 minutes before the application of an alcohol series (50–100%) then allowed to stand for 10 minutes in an H_2_O_2_ solution. After being washed with PBS, these sections were heated in a citrate buffer solution at 800 W for 4-5 minutes and allowed to stand in secondary blocker substance for 20 minutes. Each slide was allowed to stand for 75 minutes in different dilutions of the primary antibody (Anti-CPS1 at 1 *μ*g/mL and Anti-Arginase at 1/250-/500), before being stained by the Anti-Arginase (cod: ab124687, Abcam Plc., Cambridge, UK) and Anti-CPS-1 (cod: ab45956, Abcam Plc., Cambridge, UK). A diaminobenzidine solution was used as an achromogen, Mayer's hematoxylin as a counterstain for 3–5 minutes, and PBS as a negative control. The preparations were photographed after being covered with the appropriate covering materials. As a result of the immunohistochemical staining, the preparations were divided into 4 categories according to the tissue percentage of immunopositive reaction areas: mild (+), moderate (++), severe (+++), and very severe (++++). The blocked tissues were cut into 4-5 *μ*m thick sections before being stained with hematoxylin and eosin (H&E), and then the areas found to be appropriate for histopathological evaluation were photographed. These tissues were blindly evaluated by two histologists. The results of the statistical comparisons of all of the information obtained during the evaluation of the data, within the groups and between the groups, were evaluated.

### 2.6. Statistical Analyses

The results were reported as the means ± standard deviation. Data analyses were performed using the statistical software SPSS for Windows (version 13.1; SPSS, USA). The Kruskal-Wallis test was used to compare the groups. A Bonferroni adjusted Mann-Whitney *U* test was used to compare the two groups. The results are given as the mean ± SD. *P* values of <0.05 were regarded as statistically significant.

## 3. Result

### 3.1. Biochemical Parameters

The AST level of the I/R group (65.3 ± 11.5 IU/L) was strongly higher than the control group (23.3 ± 7.5 IU/L, *P* < 0.001) and the I/R+ADA group (46.7 ± 8.5 IU/L, *P* = 0.003). The AST level of the I/R+ADA group was higher than the control group (*P* < 0.001). The ALT level of the I/R group (59.2 ± 17.5 IU/L) was strongly higher than the control group (41.1 ± 11.7 IU/L, *P* = 0.010). The urea level of the I/R group (22.2 ± 9.3 mg/dL) was significantly lower than the I/R+ADA group (42.0 ± 13.8 mg/dL, *P* = 0.002) and the control group (39.8 ± 4.6 mg/dL, *P* < 0.001). The TNF-*α* level of the I/R group (430.8 ± 70.1 pg/mg protein) was significantly higher than the I/R+ADA group (338.0 ± 71.6 pg/mg protein, *P* = 0.006) and the control group (345.5 ± 53.3 pg/mg protein, *P* = 0.008). The NO level of the I/R group (8.0 ± 1.1 *μ*mol/g protein) was strongly higher than the I/R+ADA group (6.3 ± 1.2*μ*mol/g protein, *P* = 0.008) and the control group (6.5 ± 1.5 *μ*mol/g protein, *P* = 0.010). All results are shown in Table 1.

### 3.2. Histological Parameters

**Figure 1:**
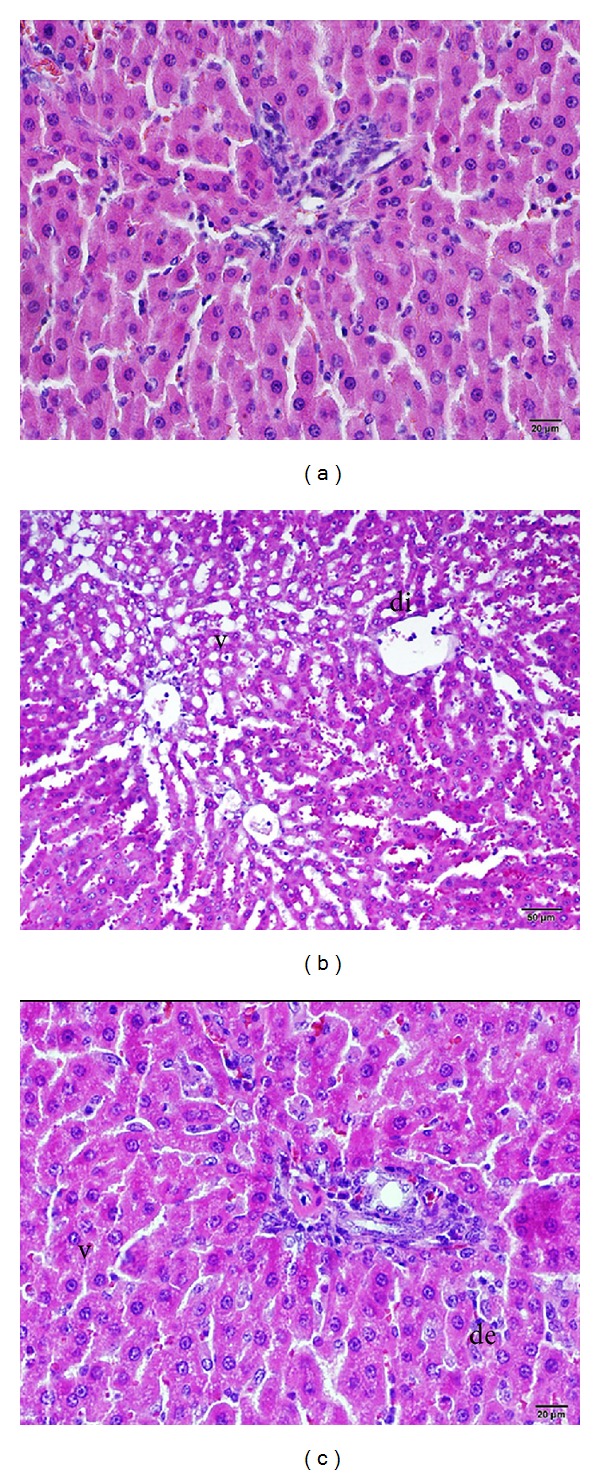
Histopathologic examination of liver tissue by light microscopy; (a) control group, (b) I/R applied group, di: dilatation and v: vacuolization, (c) I/R+ADA applied group, de: degenerative cell, H&E stain.

There were no textural or cellular deformities found in the histopathological examinations of the control livers stained using the H&E method. The morphological structures were observed to have a normal histological appearance (Figure 1(a)).

The histopathological examination of the I/R group livers stained using the H&E method revealed hepatocyte necrosis with severe cellular deformities (Figure 1(b)). A vasoconstriction was detected in the early reperfusion or acute phase with an increase in the amount of leukocytes, platelet aggregation in the sinusoids, endothelial cell swelling, cell surface recesses and protrusions, and vacuolization related to intracellular edema and neutrophilic infiltration. In the sinusoids, there were partial dilatations and Kupffer cells protruding into the lumen observed to be flat, round, and bulging. Necrotic losses related to degeneration were observed in the hepatocytes near the central vein and the area surrounding the portal vein. Acute liver capillaries in the connective tissue surrounding areas of severe congestion were seen; an increase in the number of neutrophils was found. In the acute phase there was an increase in the number of neutrophils in the connective tissue surrounding the areas of severe congestion in the liver capillaries (Figure 1(b)).

In the I/R+ADA group, the histopathological examination after the H&E staining method revealed lower tissue and cellular deformities than the I/R group (Figure 1(c)). Although the sinusoidal dilatation was decreased, there were long-course structures similar to those in the control group. The sinusoidal wall-settled Kupffer cells were observed to be more flat and stained deep basophilically (Figure 1(c)).

#### 3.2.1. Immunohistochemical Parameters

In the control group, while the surroundings of the central vein and portal area were less immunoreactive, the endothelial cells found in the dilatation areas were stained heavily positive. Although the hepatocyte nuclei of the I/R group were negatively immunoreactive, the immunoreactivity of the hepatocyte nuclei of the I/R+ADA group was observed to be very intense.

Staining of the liver tissues using the immunoperoxidase method revealed the antiliver arginase immunopositivity to be 15% (++), 55% (+++), and 30% (++++) in the control group; 65% (++), 30% (+++), and 5% (++++) in the I/R group; and 15% (++), 40% (+++), and 45% (++++) in the I/R+ADA group.

In the adult rat hepatic parenchyma of the control group, there was a heterogeneous distribution of CPS-1 in all hepatocytes, except for a narrow area around the terminal hepatic venules. There was CPS-1 reactivity observed in the hepatocyte cytoplasm and mitochondrial matrix stained using immunohistochemical methods.

Staining of the liver tissues using the immunoperoxidase method revealed the anti-CPS-1 immune positivity to be 5% (++), 30% (+++), and 65% (++++) in the control group; 50% (++), 45% (+++), and 5% (++++) in the I/R group; and 5% (++), 65% (+++), and 30% (++++) in the I/R+ADA group (Figure 3).

**Figure 2:**
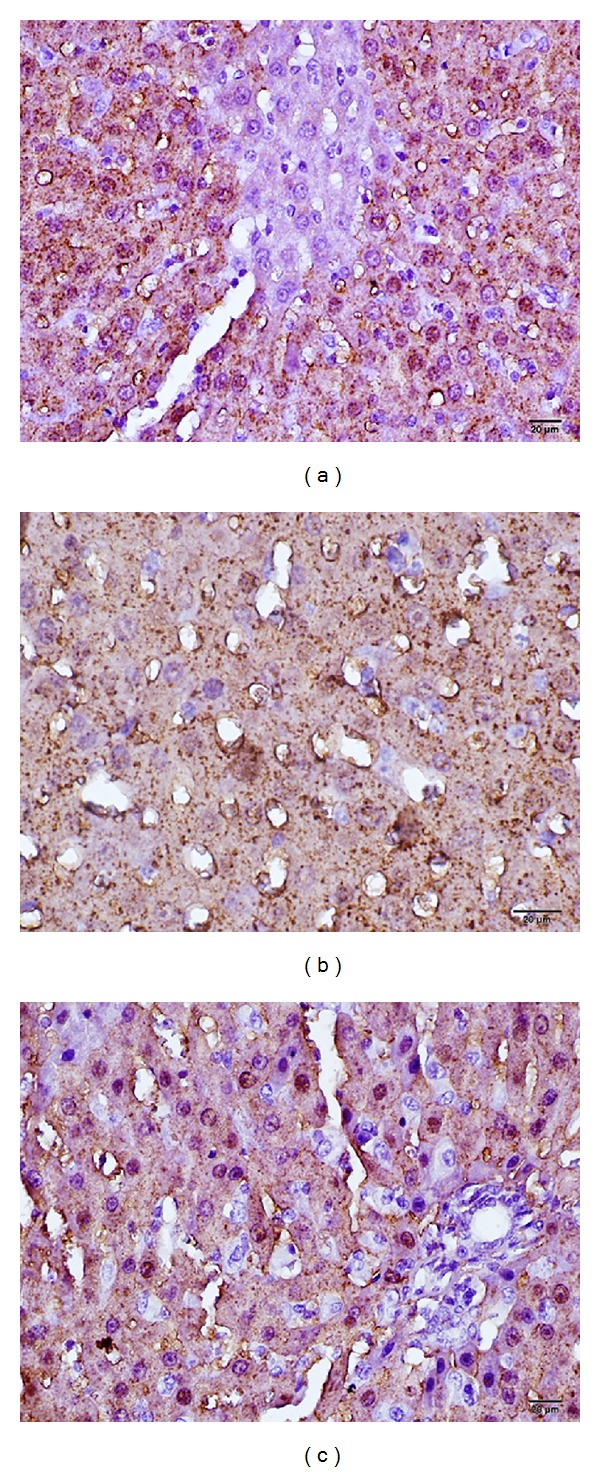
Histopathologic examination of liver tissue by light microscopy; immunohistochemical staining of liver tissues with immunoperoxidase method revealed strong and diffuse reactivity; (a) control group, (b) I/R applied group, (c) I/R+ADA applied group, immunoperoxidase stained Antiliver arginase antibody.

**Figure 3:**
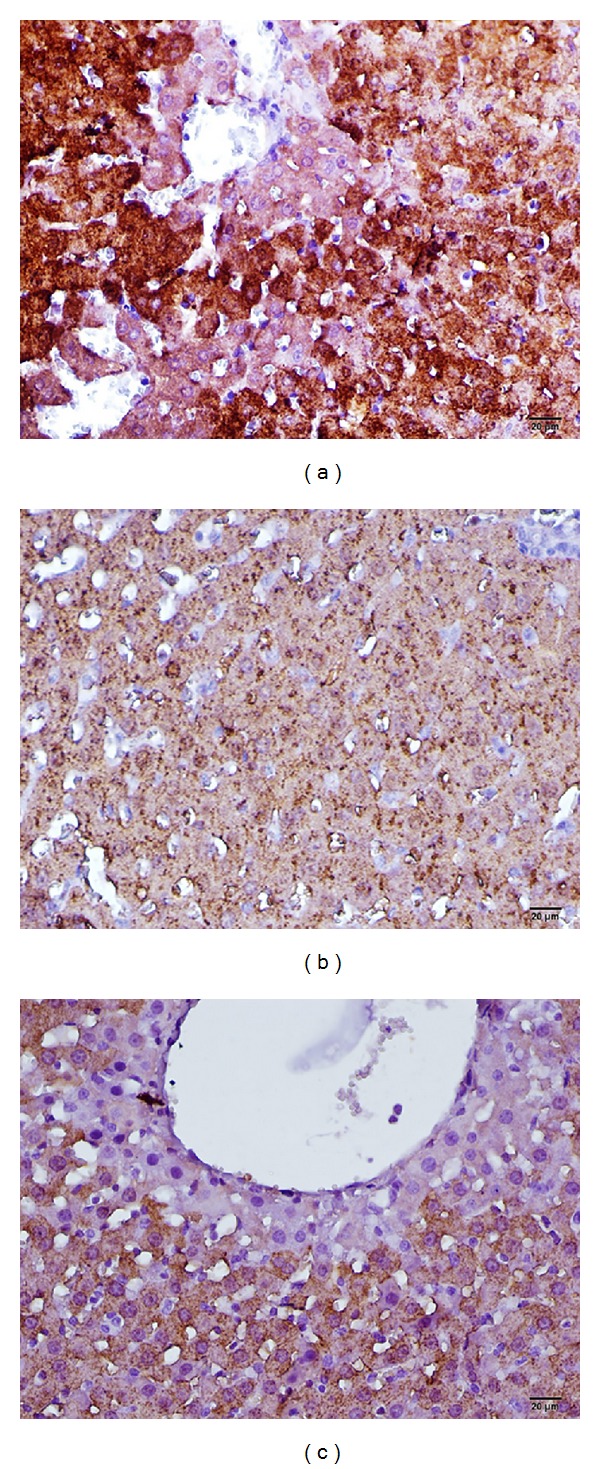
Histopathologic examination of liver tissue by light microscopy; immunohistochemical staining of liver tissues with immunoperoxidase method revealed strong and diffuse reactivity; (a) control group (b) I/R applied group, (c) I/R+ADA applied group, immunoperoxidase stained Anti-CPS1 antibody.

The arginase results are shown in Figure 2, and the CPS-1 results are shown in Figure 3. All histological results are shown in Table 2.

## 4. Discussion

In our study, the tissue TNF-*α* and NO levels of the I/R group were found to be significantly high and the serum urea level to be significantly low. The tissue TNF-*α* and NO levels of the I/R+ADA group were significantly lower than the I/R group; however, the TNF-*α*, NO, and urea levels were similar to the control group. In the tissues stained with H&E, it was observed that cell damage in the I/R group was significantly high, while it was low in the I/R+ADA group. Immunohistochemical staining of the arginase and CPS-1 activities were significantly low in the I/R group. Serum AST and ALT levels showed a significant increase during I/R. The findings of the present study have shown that two hours of aortic occlusion, followed by 2 hours of reperfusion, induce severe liver parenchymal damage.

NO plays an important and controversial role in I/R injury. Previous studies reported that overabundant NO levels induce apoptosis [21, 22]. Some other studies reported that increased NO concentration protects cells from apoptosis by vasodilatation [23, 24]. NO is synthesized from arginine and O_2_ by NO synthase (NOS) [25]. NO may mediate protection from I/R injury by the activation of the intracellular pathway, guanylate cyclase, which in turn activates protein kinases G and C, which leads to the opening of the mitochondrial ATP-dependent K+ channels [26]. A previous study has shown that the upregulation of vascular arginase inhibits NO-mediated vasodilation during I/R. In particular, the authors have demonstrated that the protein expression of arginase was augmented by I/R [27]. The inhibition of arginase activity in the I/R vessels induces the stimulated NO production, and thus, restores NO-mediated vasodilatation. An arginase inhibitor increases NO production and dilatation in normal vessels and also restores the NO-mediated dilatation after I/R [28].

The release of NO is related to oxidative stress, and previous I/R studies have shown the NO level to be high in the I/R group and low in the treatment group [21, 29]. NO-induced cell death is generally considered to be associated with DNA damage or mitochondrial damage. In the previous study, it was demonstrated that the endoplasmic reticulum stress pathway was involved in NO-mediated apoptosis [30]. Previous studies reported that infliximab therapy decreased serum NO levels [31]. Similarly, current study showed that ADA treatment diminished tissue NO levels during I/R injury. In our study, the NO and TNF-*α* levels of the I/R group were higher than in the control and I/R+ADA groups. The examination of the histological preparations (H&E) revealed many findings of cell degeneration in the I/R group. ADA treatment may ameliorate damage of I/R injury.

CPS-1 and arginase are key enzymes in the urea cycle [32]. Arginase exists in two isoforms, liver-type arginase I and nonhepatic-type arginase II. The former is a cytosolic enzyme found primarily in the liver [33]. CPS-1 is a liver-specific, intramitochondrial, rate-limiting enzyme in the urea cycle, which plays a staminal role in protein and nitrogen metabolism. Previous studies have shown that a quantitative change in this enzyme's expression and function can affect NO production by limiting substrate availability [34]. It was reported that inhibiting the degradation of arginine by arginase and CPS-1 increases NO synthesis [28, 35], so that the tissues of the I/R group were preserved. Therefore, decreased activity of arginase is a potential factor that excessive NO levels [36]. In our study, low levels of urea and increased NO in the I/R group indicate the inhibition of arginase and CPS-1.

In the current study, arginase and CPS-1 activities were low in the I/R group. Due to the activation of the NO synthesis pathway from the arginine in this group, we have found the NO level to be high in the control group and I/R+ADA group. The NO level, which was also higher than in the control group, was higher than that required for the basal body level, suggesting that NO triggers cell damage, rather than tissue protection, via the vasodilatation effect. The results of the I/R+ADA group were similar to the control group. ADA treatment protects the tissues from I/R damage by providing maintenance of the body's equilibrium state of the oxidative stress mechanism.

NO bioavailability may be critically regulated by arginase by competing with NOS for their extensive substrate L-arginine [37]. Arginase, CPS-1, and NOS are immensely important for maintaining the delicate balance in the organism [38]. ADA treatment during I/R may maintain the balance between arginase and NOS, prevent excessive NO release by decreasing both arginase and CPS-1 activities, and prevent NO related vasodilatation by increasing both arginase and CPS-1 activities. Therefore, this treatment reduces NO formation through the suppression of NOS expression in liver I/R. The I/R+ADA group may be preserved from cell cytokines and ROS-mediated apoptosis caused by NO. Previous study reported that NO levels may be increase during I/R; however, NO-mediated vasodilatation functions are inhibited by increased H_2_O_2_ due to ROS in I/R [6]. We speculate that arginase and CPS-1 may be downregulated by increased NO level during I/R and their tissue levels may be decreased. Therefore, increased NO levels may lead to cell damage by oxidative stress rather than vasodilatation in I/R. ADA may maintain this balance.

Various factors are involved in I/R injury, including ROS production, calcium overload, neutrophilic infiltration, and cytokine release. The destructive effects of I/R result from the generation of ROS, subsequent to reoxygenation, that causes direct tissue damage and initiates a cascade of destructive cellular responses, leading to inflammation, cell death, and organ damage [39, 40]. Among these mediators, TNF-*α*, which plays a key role in the inflammatory reaction, is thought to play a major role in I/R injury. High TNF-*α* increases ROS, causing increased apoptosis [41]. It has been demonstrated by some studies that a prophylactic anti-TNF-*α* treatment, such as infliximab, may be an effective therapeutic strategy for preventing I/R-induced injury [18, 42, 43]. ADA is a potent antibody against TNF-*α*, which can neutralize all forms (extracellular, transmembrane, and receptor-bound) of TNF-*α*. In this study, the issue TNF-*α* level of the I/R group was significantly higher than the control and I/R+ADA groups. It was observed that the TNF-*α* level of the I/R+ADA group was similar to the control group.

The increase of AST and ALT observed in I/R group can be explained by the hepatocyte damage which is caused by the ROS and cytokines during the I/R phase. The lower increase of AST and ALT levels was observed in animals of I/R+ADA group when compared to I/R group. Our results have shown that ADA may have protective effects against liver I/R injury, because of its anti-inflammatory and antioxidant properties, which reduce TNF-*α* release. The studies of ADA in rats were limited, and it has been reported that the maximum serum level of ADA was reached after an average of five days, after subcutaneous administration in humans [44]. In the current study, we administered the ADA intraperitoneally five days before I/R, and we have shown it to be protective in I/R.

## 5. Conclusion

During I/R, arginase and CPS-1 enzymes are excessively inhibited; therefore, excessive NO synthesis and the accompanied cytokine increases have been shown to cause apoptosis. Treatment with ADA as an inhibitor of TNF-*α* during I/R decreases cytokines, prevents the increase and decrease of NO by maintaining the balance between arginase, CPS-1, and NOS, and consequently protects the cells from death.

## Figures and Tables

**Table 1:** All the biochemical results of three groups.

	Control (mean ± sd)	I/R (mean ± sd)	I/R + ADA (mean ± sd)
ALT (IU/L)	41.1 ± 11.7	59.2 ± 17.5^F^	51.4 ± 8.8
AST (IU/L)	23.3 ± 7.5	65.3 ± 11.5^∗,¶^	46.7 ± 8.5*
Creatinine (mg/dL)	0.4 ± 0.05	0.5 ± 0.05	0.5 ± 0.07
Urea (mg/dL)	39.8 ± 4.6	22.2 ± 9.3^∗,W^	42.0 ± 13.8
TNF-*α* (pg/mg protein)	345.5 ± 53.3	430.8 ± 70.1^X,*α*^	338.0 ± 71.6
NO (*µ*mol/g protein)	6.5 ± 1.5	8.0 ± 1.1^F,†^	6.3 ± 1.2

ADA: adalimumab; I/R: ischemia/reperfusion; TNF-*α*: tumor necrosis factor-alpha; NO: nitric oxide.

For ALT: ^F^ *P* = 0.010 versus control group.

For AST: *P < 0.001 versus control group; ^¶^ *P* = 0.003 versus I/R + ADA group.

For Urea: *P < 0.001 versus control group; ^W^ *P* = 0.002 versus I/R + ADA group.

For TNF-*α*: ^X^ *P* = 0.008 versus control group; ^*α*^ *P* = 0.006 versus I/R + ADA group.

For NO: ^F^ *P* = 0.010 versus control group; ^†^ *P* = 0.008 versus I/R + ADA group.

**Table 2:** Histopathologic examination of liver tissue.

	Sinusoid dilatation (mean ± sd)	Hepatocyte degeneration (mean ± sd)	Neutrophil infiltration (mean ± sd)	Antiliver arginase reactivity (mean ± sd)	Anti-CPS-1 reactivity (mean ± sd)
Control	0 ± 0	0 ± 0	0 ± 0	3.0 ± 0.8	3.8 ± 0.4
I/R	3.0 ± 0.4^∗,¶^	3.1 ± 0.3^∗,A^	2.8 ± 0.8^∗,W^	2.1 ± 0.8^¥,B^	2.0 ± 0.6^∗,¶^
I/R + ADA	2.2 ± 0.8*	2.2 ± 0.7*	1.2 ± 0.4*	3.7 ± 0.6	3.0 ± 0.8^X^

CPS 1: carbamoyl phosphate synthetase 1; I/R: ischemia/reperfusion; ADA: adalimumab.

For sinusoid dilatation: *P < 0.001 versus control group; ^¶^ *P* = 0.015 versus I/R + ADA group.

For hepatocyte degeneration: *P < 0.001 versus control group; ^A^ *P* = 0.005 versus I/R + ADA group.

For neutrophil infiltration: *P < 0.001 versus control group; ^W^ *P* < 0.001 versus I/R + ADA.

For Antiliver arginase reactivity: ^¥^ *P* = 0.011 versus control group; ^B^ *P* = 0.002 versus I/R + ADA.

For Anti-CPS1 reactivity: *P < 0.001, ^X^ *P* = 0.015 versus control group; ^¶^ *P* = 0.015 versus I/R + ADA group.
